# A Scoping Review of Financial Elder Exploitation Interventions

**DOI:** 10.1093/geroni/igab046.1254

**Published:** 2021-12-17

**Authors:** Stephanie Skees

**Affiliations:** Washington University in St. Louis, Saint Louis, Missouri, United States

## Abstract

Elder financial exploitation (EFE), defined by the National Center for Elder Abuse (2021) as “the misappropriation of an older person’s money or property,” is a continuing public health crisis shown to cost individuals at least $2.9 billion a year (MetLife Mature Market Institute, 2011). Many believe this impact will increase exponentially due to the effects of COVID-19. In fact, a recent study conducted by Chang & Levy (2021) found that the prevalence of elder abuse as a whole increased from 1 in 10 older adults to 1 in 5 in the past year. Although increased collaboration between state attorneys general, Adult Protective Services, and financial institutions has driven progress in the field; there is still little known regarding EFE interventions. To address this issue, this study conducts a scoping review of the EFE intervention literature. This approach was chosen over a systematic review primarily due to the lack of a universal definition of EFE, as well as the limited number of studies available delineating between EFE and elder abuse as a whole. The main findings of the review reveal that current EFE intervention practices are focused on preventing abuse before it occurs by addressing risk factors for abuse in older adults; and are largely reliant on Adult Protective Services and the legal system. This finding is significant because state policies differ in their qualifications of EFE, thus leaving many older adults vulnerable and unprotected. Further interventions that address EFE while it is occurring and alignment across governing bodies are needed.

